# Propolis as a Remedy for Diarrhœas, Acute and Chronic

**Published:** 1867-09

**Authors:** H. O. Hitchcock

**Affiliations:** Kalamazoo, Mich.


CHICAGO MEDICAL JOURNAL.

VOL. XXIV. SEPTEMBER, 1867. No. 9.

### Original Contributions.

### PROPOLIS AS A REMEDY FOR DIARRHŒAS, ACUTE AND CHRONIC.

Read before the Kalamazoo Valley Medical Association, October, 1866.

By H. O. HITCHCOCK, M.D., Kalamazoo, Mich.

I take pleasure in bringing to the notice of the members of this Society an article whose medicinal properties have not, as yet, I believe, been laid before the profession. I am confident that, on thorough and honest trial, it will be found to possess virtues that make it worthy a place in our pharmacopœia.

Sometime during the last spring, (1866,) a gentleman made some statements to me in relation to the efficacy of a certain article in diarrhœas, acute and chronic. He claimed for it, also, great efficiency in the treatment of dysentery, and also in cholera.

I promised to give it a fair trial as soon as any cases indicating its use should present themselves. The article then brought to my notice is thus alluded to, and briefly described, in vol. iii of the New American Encyclopedia, under the article "Bee," viz.: "Wax is not the only material used by bees in their architecture; besides this they employ a reddish-brown, odoriferous, glutinous resin, more tenacious and extensible than wax, called *propolis*, which they obtain from the buds of the poplar and birch, and from various resinous trees."

During the past summer I have prescribed it as a medicinal remedy in numerous cases of acute and chronic diarrhœa and also in several cases of dysentery. I have also presented samples of it to several members of the Society, requesting them to give it a fair trial, and note faithfully its efficacy.

In my own experiments with it, it has proved one of the best and most reliable remedies I have used, in nearly all cases of simple mucous diarrhœa, even when violent, and accompanied by severe pains, vomiting, and collapse. In many cases a single dose has been all that was required. In several cases of diarrhœa in children it has acted like a charm. It appears to possess an anodyne and soporific property, and yet it does not cause constipation of the bowels, but brings them to a normal action at once.

In several cases of dysentery, when given early, it has appeared to have a marked effect in arresting the disease. But in cases where the disease had become established, and especially in those cases where malarious congestion of the mucous membrane characterized the disease, it has appeared nearly worthless.

I have prescribed it in several cases of chronic diarrhœas, in some of which the disease was contracted in camp. All of these patients have reported to me "that the medicine has acted like a charm," being far more efficacious than any other medicine they have ever used.

The experiments of Drs. Porter, Fisk, and Southard, of this village, have fully corroborated my own. Below I have given several cases.

The propolis is a green resin of a dark-reddish, or yellowish-brown color, of a glistening fracture, slightly conchoidal, of an aromatic taste and smell, entirely insoluble in water, and nearly so in ether, but readily soluble in alcohol and in liquor potassæ. At first I used the tincture, which is of a beautiful wine color.

The following is the formula by which I prepared it for use:

℞.—Selected propolis, ---------------------------- ʒij.

Alcohol, ------------------------------------------ ℥iv.

Dissolve.

Dose for an adult, ½ to 1 teaspoonful in sweetened water; for a child, 5 to 20 or 30 drops, after each stool.

When the alcoholic solution is mixed with water or simple syrup, there is a copious deposit thrown down. The taste is pungently aromatic, but is not unpleasant even to children.

Lately I have used an alkaline solution, as follows:

℞.—Propolis, ---------------------------------------- ℥ji.

Liq. potassæ, ------------------------------------ ℨi.

Solve *et adde* aq., simpl. syrup, partes āā ℥ij.

Dose ½ a teaspoonful after each stool.

The advantage of the alkaline solution is that its admixture with water or simple syrup does not cause a deposit.

Case I. G. S., a man 72 years of age, called me to see him in the middle of the night, Nov. 14, 1866. He had the day before drank a glass or two from a cask of recently manufactured beer; otherwise he had taken nothing unusual, and appeared at bed time in his usual health. I found him vomiting and purging very frequently—vomiting an ashy looking substance, with dirty, green floccules in it, and the stools were mostly of a thin substance like rice water. There was great pain at the epigastrium and throughout the abdomen, with decided and very troublesome cramps in the legs. Skin was cold and clammy, except about the head; the eyes were sunken; the pulse very feeble, scarcely to be felt at the wrist. I gave him a teaspoonful of a saturated tincture of propolis, and nothing else, except to apply extremely dry heat. He did not vomit again, but had a stool in about 15 minutes. This was followed by another teaspoonful of the medicine. His pain now began to subside, the pulse to be less in frequency, but fuller and stronger. In about an hour he had another stool, which I followed by a dose of ½ a teaspoonful of the remedy. He required no more, and became speedily convalescent.

Case II. I was consulted about this time by a young man for a friend of his, who had gradually increasing on him for some months a chronic diarrhœa, accompanied by pain and soreness throughout the abdomen. I gave him the alkaline solution above indicated, in doses of ½ a teaspoonful three times a day. Three days after, the pain and soreness had all passed away, and the stools were nearly or quite normal in frequency and consistence.

CASE III. (By Dr. Porter.) H. P., during a typho-malarious fever, was attacked with diarrhœa. Dejections every 20 or 30 minutes; very dark and watery, and accompanied by vomiting. Ordered a teaspoonful of a mixture of equal parts of tinct. propolis, and simple syrup, to be repeated every two or three hours. The first two doses were rejected. After the third, vomiting ceased, and no stools for two hours, and another dose ended the diarrhœa entirely.

CASE IV. (By Dr. Fish.) October 31, 1866, was called to see Mrs. P., who had had chronic diarrhœa for two years, not having had a natural stool within that period; stools generally were from to two to four per day. There was attending the diarrhœa a great deal of soreness over the liver, stomach, and bowels. In one week the tincture of propolis, in doses of ½ a teaspoonful three times a day, had entirely cured her.

I take pleasure in submitting the above to the profession, and in submitting the article to, and asking for it, the careful and honest test of its experience.

